# Intranasal Midazolam With Lidocaine for Sedation in Pediatric Myringotomy and Tube Surgery: A Randomized Controlled Trial

**DOI:** 10.7759/cureus.27181

**Published:** 2022-07-23

**Authors:** David A Ullman, Jennifer M Victory, Melissa B Scribani

**Affiliations:** 1 Anesthesiology, Bassett Healthcare Network, Columbia University College of Physicians and Surgeons, Cooperstown, USA

**Keywords:** nasal intranasal mucosal atomization device, myringotomy, atomized sedation, pediatric anesthesia, intranasal administration, anesthesia

## Abstract

Background and Aims: Intranasal midazolam (INM) sedation for children has been associated with side effects. This prospective, double-blind, placebo-controlled trial assessed whether the addition of lidocaine to INM (INM+L) affected efficacy or discharge time among pediatric patients undergoing elective bilateral myringotomy and tube placement (BMT).

Methods: This trial enrolled children aged between 18 months to seven years undergoing BMT, physical status class 1 or 2, in a single academic medical center. Interventions were placebo (intranasal saline), INM only (0.2mg/kg of INM concentration 5mg/ml), and INM+L (0.2mg/kg INM with addition of lidocaine 4% based on 25% of midazolam volume). Outcomes included post-anesthesia care unit times, observed behavioral distress (OBD) visual analog scale (VAS) (by nurse and parent), and sedation scores by certified registered nurse anesthetist (CRNA) and registered nurse (RN).

Results: Forty-two subjects were included, 14 in each group, with 52% female, 41% physical status 2, and an average age of 2.7 years. Post-anesthesia care unit times averaged 36.5 minutes (range 15-132 minutes), with no delay in discharge with INM or INM+L versus placebo (p=0.88). Verbal complaints were highest among INM+L at the time of administration (p=0.01). RN-scored OBD at one minute post administration differed significantly across the three groups (p=0.01). Parental OBD scores did not differ across treatment groups. Agitation was greatest at time of induction of anesthesia in the placebo group (p=0.01).

Conclusions: The addition of licodaine to INM does not adversely influence time to discharge and does not reduce side effects, improve efficacy, or change duration of action of INM.

## Introduction

The ideal premedication for pediatric patients remains controversial. Peri-operatively, an agitated child is at risk for undesirable sequelae such as injury, anxiety, and postoperative sleep disorders [1-5]. An ideal preanesthetic medication should reduce anxiety and allay parental separation fears while minimizing any delay in discharge [6]. Untoward side effects should be minimized, such as painful intramuscular injection or delayed onset via oral/rectal administration. Crying after intranasal midazolam (INM) administration has been described as a causative measure of discomfort from the drug [7], as well as nasal burning, bitter aftertaste [8], and mild epistaxis [9]. A potential pitfall with orally administered midazolam is low bioavailability [10]. The bitter aftertaste is also detrimental to patient compliance and is cause for rejection of oral or sublingual administration of midazolam [7,10] resulting in reduced efficacy. Other concerns for orally administered midazolam include possible adverse effects on gastric pH and gastric volume [11]. 

Administration of INM with the addition of lidocaine (INM+L) in an attempt to reduce side effects has been described [8,12]. For years, the lead author has utilized readily available components at the bedside for INM+L administration in a single atomization versus two separate atomizations in an effort to reduce patient discomfort. The current study was designed to investigate whether the simple, convenient combination of lidocaine and midazolam could improve patient compliance and preoperative sedation in a timely and safe fashion while reducing unwanted side effects in pediatric patients, aged between 18 months to seven years, scheduled for bilateral myringotomy and tube placement (BMT). All cases were performed under mask general anesthesia in a teaching hospital as outpatients. A secondary objective was to examine discharge times from the post-anesthesia care unit (PACU) in patients receiving INM alone, INM+L, or placebo.

This work was previously presented as a poster at the International Anesthesia Research Society (IARS) Annual Meeting in Montreal, Canada, on May 19, 2019.

## Materials and methods

Ethical approval for this study was provided by the Mary Imogene Bassett Hospital Institutional Review Board (IRB), Cooperstown, New York, United States, on January 6, 2015. This study was conducted in compliance with the ethical standards of this IRB as well as with the Helsinki Declaration. Written informed consent was obtained from the parents or legally authorized representatives of the pediatric patients. This study was registered prior to subject enrollment (on February 2, 2015) with clinicaltrials.gov, trial# NCT02356705 (Registered Trial Name: Intranasal Midazolam in Children as a Pre-Operative Sedative - Part 2).

**Figure 1:**
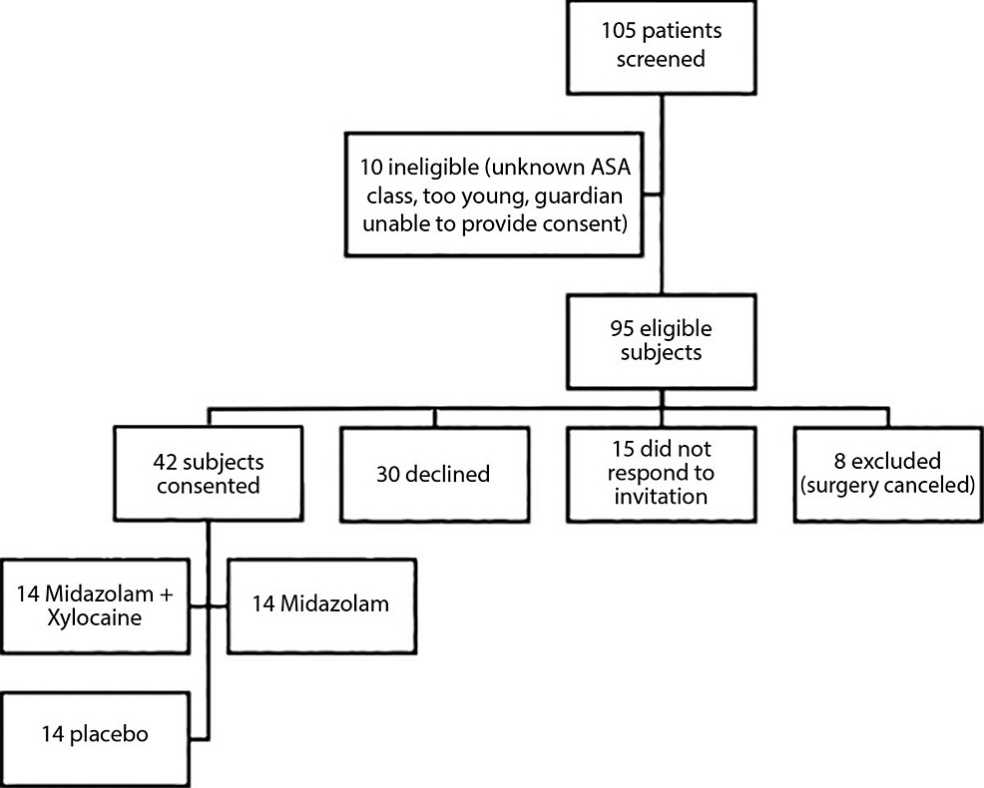
Flow diagram of included and excluded subjects. ASA: American Society of Anesthesiologists Xylocaine is a brand name for lidocaine

This study was prospective, double-blind, randomized, and placebo controlled. Eligible subjects were children aged 18 months to seven years, scheduled for BMT requiring mask anesthesia at Bassett Medical Center between February 2015 and December 2017. All patients were seen in our ENT clinic and diagnosed with chronic bilateral otitis media and noted to be surgically naïve. Eligible subjects had to be classified as physical status (PS) Class 1 or 2 according to the American Society of Anesthesiologists. Furthermore, subjects were only eligible if the parent (or legally authorized representative) was willing and able to provide informed consent and had the ability to complete the behavioral observed distress (OBD) visual analog scale (VAS). Children with physical status (PS) Class 3 or greater, with a history of allergy to midazolam or lidocaine, or presence of acute respiratory infection at the time of surgery, were excluded (Figure 1).

Eligible, consented study participants were randomly assigned to one of three treatment groups in a 1:1:1 ratio. The treatment groups were:

Group 1 - Placebo, patients received intranasal saline Group 2 - INM, patients received nasal midazolam only (0.2 mg/kg of INM; midazolam concentration 5mg/ml) Group 3 - INM+L, patients received 0.2 mg/kg INM and lidocaine 4% in a dose based on 25% of the volume of the calculated midazolam dose; midazolam concentration 5mg/ml. For example, a 20kg child would have received 4mg midazolam (0.8ml). Thus, 0.2ml of lidocaine 4% would be added to the midazolam for a 1.0ml total volume.

**Figure 2:**
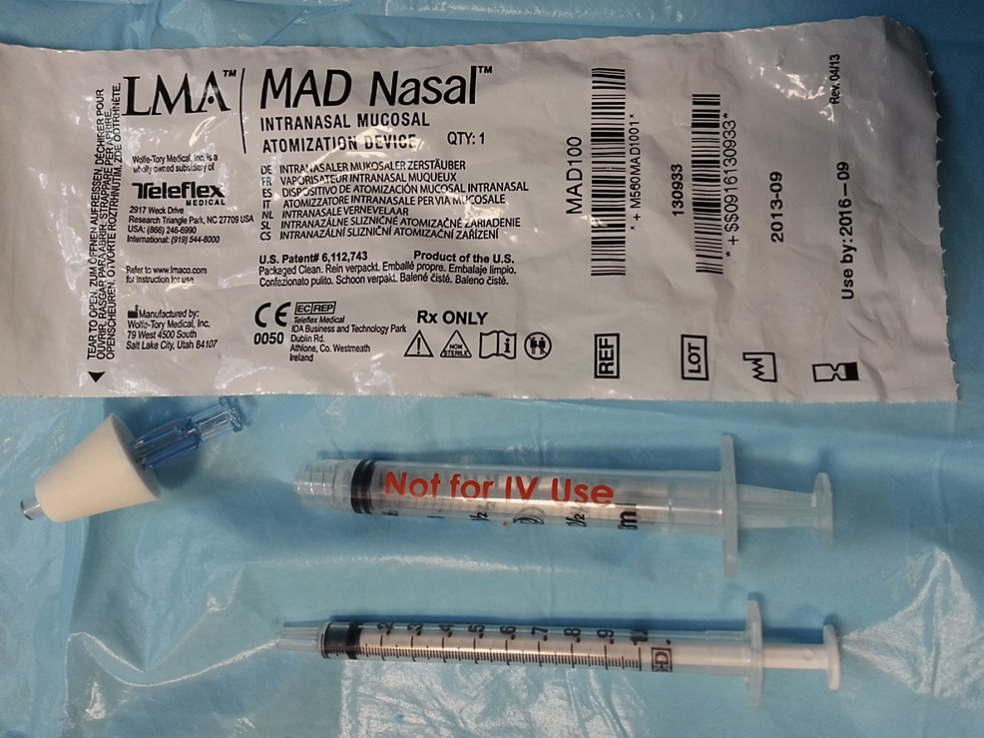
Intranasal Mucosal Atomization Device used for drug administration

All individuals involved in the execution of the study, including investigators and administering staff, were blinded with the exception of the inpatient pharmacist. Preparation of the study drugs was carried out by this pharmacist. The drugs were prepared in two vials: midazolam/placebo and lidocaine/placebo, with specific instructions directing the pharmacist on how much to withdraw from each vial and mix in one syringe. Syringes were 3cc or 1cc (tuberculin) depending on the size of the dose. The volume of medication was shielded from investigators and administering staff to preserve blinding using tape around the syringe and was then sent to the RN who would deliver the dose to the subject. The medication was administered into a naris using the LMA MAD Nasal™ Intranasal Mucosal Atomization Device (Teleflex Incorporated, Wayne, Pennsylvania, United States) (Figure 2). 

All patients had mask anesthesia, provided by a staff CRNA under the medical direction of a staff anesthesiologist with no additional analgesia administered in the PACU. All patients received a standardized mask anesthetic administration and maintenance consisting of nitrous oxide combined with either sevoflurane or desflurane. Acetaminophen 10mg/kg was administered per rectum after administration of anesthesia.

The same administering RN enrolled patients and collected informed consent documentation from the parents pre-operatively in the ENT clinic. This RN collected the following data points within 10 minutes of transfer to the operating room: verbalized complaints at the time of study drug administration, one minute and five minutes post-administration; any episodes of epistaxis; OBD VAS at one minute and five minutes post-administration; sedation score [13] at 10 minutes and 15 minutes post-administration (measured as: agitated, alert, calm, drowsy, asleep). Accompanying parents/legally authorized representatives completed the OBD VAS at one minute and five minutes post-administration. A certified registered nurse anesthetist (CRNA) assigned to the study room each day completed the sedation scale at the time of transport to the operating room and at the time of induction of anesthesia. 

For the OBD VAS measures, the RN and accompanying parent were asked to “place a mark on the line to indicate how distressed you think the child is right now”, with “0” indicating no distress and “10” indicating the most distress possible. One additional endpoint included time from the PACU using Aldrete's scoring system of at least 9 points, to discharge home.

The randomization schedule was produced by the study statistician using SAS software, Version 9.3 (2011; SAS Institute Inc., Cary, North Carolina, United States) with a randomized block design in blocks of random permutations of the numbers one, two, and three. Based on pilot data, collected from February 2012 to January 2014, it was estimated that 15 subjects in each treatment group would provide adequate statistical power (power=0.824, two-tailed alpha of 0.05) to detect a two-unit difference (estimated standard deviation=3.3 units) in RN-observed OBD VAS scores at one-minute post-administration. Due to limitations in the number of providers (attrition of one otolaryngologist) and low numbers of willing eligible patients during the two-year study period, recruitment was halted once 14 subjects were recruited in each treatment arm. This did not amount to a significant drop in statistical power (power=0.793). 

Comparisons between the three treatment groups were carried out using chi-square for categorical outcomes (e.g., verbalized complaints, sedation scores). The Kruskal-Wallis test was used to compare VAS scores and age across the three treatment groups. For three-group comparisons with an overall p<0.05, pairwise relationships were explored by first converting the data to ranks and then performing Scheffe’s post hoc tests via parametric analysis of variance (ANOVA). Probabilities of less than 0.05 were considered statistically significant. All analyses were conducted using SAS 9.3.

Pre-screening identified 105 pediatric subjects scheduled for BMT. Among those, 10 were excluded due to ineligibility (unknown PS class, too young, guardian unable to give consent). Among 95 eligible subjects, eight had surgeries canceled and were therefore excluded. Of the 87 remaining eligible subjects, 15 parents/guardians did not respond to the invitation to participate in the study, while 30 parents/guardians declined participation. A total of 42 subjects were included in this study, 14 in each of the INM alone, INM+L, and placebo groups (Table 1). The study sample was 52% (N=22) female, 41% (N=17) PS Class 2, and 2.7 years old on average with a range of 18 months to 7 years. There were no significant differences in these characteristics across treatment groups (Table 1). 

**Table 1:** Demographic profile of participants, compared between midazolam, midazolam+lidocaine, and placebo groups (n=42) n: number; SD: standard deviation; IQR: interquartile range

	Total sample (n=42)	Midazolam Alone (n=14)	Midazolam + Lidocaine (n=14)	Placebo (n=14)	Probability
Male	20 (48%)	9 (64%)	4 (29%)	7 (50%)	0.16
Female	22 (52%)	5 (36%)	10 (71%)	7 (50%)	
Physical Status 1	25 (59%)	9 (64%)	7 (54%)	8 (57%)	0.85
Physical Status 2	17 (41%)	5 (36%)	6 (46%)	6 (43%)	
Age, mean (SD)	2.7 (1.6)	3.1 (1.5)	2.5 (1.7)	2.4 (1.7)	0.53
Median (IQR)	2.0 (1.0,3.0)	2.0 (2.0,5.0)	2.0 (1.0,3.0)	2.0 (1.0,3.0)	
Range	1-7	1-5	1-6	1-7	

## Results

There was no delay in discharge with INM or INM+L as compared to placebo (p=0.88). Patients stayed in the post-operative care unit for an average of 36.5 minutes (range 15-132 minutes). The upper limit range of 132 minutes was unrelated to the study drugs and was due to family factors. The average time from administration of the study drug to induction of anesthesia was 22 minutes (range 12-87 minutes). There was no difference in time from drug administration to induction across treatment groups (p=0.19, Table 2).

**Table 2:** Comparison of discharge times, patient complaints, observed behavioral stress, and sedation scores across treatment groups ‡ times in row headings refer to time in minutes from study drug administration n: number; SD: standard deviation; IQR: interquartile range; RN: registered nurse; CRNA: certified registered nurse anesthetist; OR: operating room

	Total sample (n=42)	Midazolam Alone (n=14)	Midazolam + Lidocaine (n=14)	Placebo (n=14)	Probability
Minutes, post-op to discharge, mean (SD)	42.1 (24.0)	44.9 (31.7)	41.6 (18.1)	39.6 (21.8)	0.88
Median (IQR)	36.5 (29.0,45.0)	39.5 (22.0,50.0)	35.5 (31.0,45.0)	35.0 (29.0,44.0)	
Range	15-132	15-132	20-90	20-109	
Minutes, drug admin to induction, mean (SD)	22.3 (11.8)	21.2 (4.9)	23.1 (18.6)	22.6 (8.1)	0.19
Median (IQR)	20.0 (18.0,22.0)	21.0 (19.0,23.0)	18.0 (17.0,20.0)	20.0 (18.0,22.0)	
Range	12-87	14-35	12-87	17-46	
Verbal complaint, administration	18 (46%)	6 (43%)	9 (82%)	3 (21%)	0.01
Verbal complaint, 1 minute^†^	9 (22%)	5 (38%)	4 (31%)	0	0.03
Observed Behavioral Distress Scores					
Parent score, 1 minute, mean (SD)	0.8 (1.9)	1.4 (2.9)	0.7 (1.3)	0.3 (0.7)	0.20
Median (IQR)	0.2 (0,0.5)	0.3 (0,0.5)	0.3 (0.1,0.5)	0.01 (0,0.3)	
Range	0-9.2	0-9.2	0-4.8	0-2.1	
Parent score, 5 minutes, mean (SD)	0.1 (0.3)	0.2 (0.4)	0.1 (0.2)	0.1 (0.4)	0.59
Median (IQR)	0 (0,0.1)	0.01 (0,0.2)	0 (0,0.05)	0 (0,0.01)	
Range	0-1.6	0-1.6	0-0.6	0-1.4	
RN score, 1 minute, mean (SD)	0.9 (2.0)	1.6 (3.0)	0.9 (1.6)	0.1 (0.1)	0.01
Median (IQR)	0.1 (0.01,0.4)	0.3 (0.1,0.6)	0.4 (0.01,0.6)	0.02 (0,0.1)	
Range	0-9.5	0-9.5	0-6	0-0.4	
RN score, 5 minutes, mean (SD)	0.2 (1.1)	0.6 (1.9)	0.02 (0.1)	0.1 (0.2)	0.08
Median (IQR)	0 (0,0.04)	0.02 (0,0.2)	0 (0,0.01)	0 (0,0.02)	
Range	0-7	0-7	0-0.2	0-0.6	
Sedation Scores					
10 minutes post-administration (RN)					0.09
Agitated	0	0	0	0	
Alert	22 (52%)	9 (64%)	4 (29%)	9 (64%)	
Calm	19 (45%)	4 (29%)	10 (71%)	5 (36%)	
Drowsy	1 (2%)	1(7%)	0	0	
15 minutes post-administration (RN)					0.01
Agitated	3 (7%)	0	0	3 (21%)	
Alert	15 (37%)	4 (29%)	3 (23%)	8 (57%)	
Calm	22 (54%)	9 (64%)	10 (77%)	3 (21%)	
Drowsy	1 (2%)	1 (7%)	0	0	
At OR Transport (CRNA)					0.39
Agitated	7 (17%)	1 (7%)	1 (7%)	5 (36%)	
Alert	7 (17%)	3 (21%)	3 (21%)	1 (7%)	
Calm	26 (62%)	9 (64%)	9 (64%)	8 (57%)	
Drowsy	2 (5%)	1 (7%)	1 (7%)	0	
At induction of anesthesia (CRNA)					0.01
Agitated	20 (48%)	3 (21%)	6 (43%)	11 (79%)	
Alert	5 (12%)	1 (7%)	3 (21%)	1 (7%)	
Calm	16 (38%)	10 (71%)	4 (29%)	2 (14%)	
Drowsy	1 (2%)	0	1 (7%)	0	

Patient discomfort, as measured by verbal complaints, was highest among the INM+L group at the time of administration (N=9,82%) compared to the INM group (N=6,43%) or placebo (N=3,21%) (p=0.01, Table 2). At one minute post administration, verbal complaints were reduced among the INM+L group and INM alone group but were still significantly more frequent than in the placebo group (p=0.03). There were no verbal complaints reported at five minutes post-administration in any of the three groups.

At one minute post administration, OBD scored by the RN observer differed significantly across the three groups (p=0.01, Table 2). Pairwise comparisons showed statistically significantly lower observed distress scores among the placebo group as compared to INM (p=0.03). The OBD score in the INM+L group was not statistically significant compared to the placebo group (p=0.05). At five minutes post administration, the RN-scored OBD was higher among the INM group versus placebo, but not significantly so (p=0.08). Parental OBD scores were not statistically significantly different across treatment groups at either one minute or five minutes post administration (p=0.20 and p=0.59, respectively).

Sedation scores were generally more favorable among the INM group at the time of induction of anesthesia, with the most agitation observed in the placebo group (p=0.01). At 15 minutes post administration, agitation was documented in the placebo group (21% of patients) whereas no agitation was seen in either INM or INM+L groups. No statistically significant differences were seen across treatment groups related to sedation scores at the time of transport to the operating room or 10 minutes post-administration (Table 2). 

## Discussion

The use of intranasal premedication was described by Wilton et al. in 1988 [13]. The dose utilized for INM of 0.2 mg/kg has been previously described [10,13]. Chiaretti et al. described the combination of INM+L to be efficacious in children while reducing side effects [8] but, unfortunately, it was not a controlled or blinded study. In 2008, Manley et al. reported using a standardized intranasal preparation of midazolam 40 mg/ml and lidocaine 20 mg/ml prepared exclusively by their pharmacy [12].

We utilized readily available and inexpensive drugs easily prepared at the bedside for this study. When utilizing INM it is critical to keep the nebulized volume as low as possible to avoid excess drug being introduced into the hypopharynx or gastrointestinal tract [14]. We utilized midazolam 5 mg/ml and preservative-free lidocaine 4% to achieve a final concentration of midazolam 0.2 mg/kg and a volume of lidocaine equivalent to 25% of the volume of the midazolam volume for a final nebulized mixture.

The use of aerosolized INM has been well described using the mucosal atomization device (MAD) to improve bioavailability while reducing the amount of drug introduced into the gastrointestinal tract [7,12]. One notable disadvantage of oral and INM "syringe only" administration is that patients have been reported to spit out the administered drug thereby reducing available drug [7]. Karl et al. reported this phenomenon with lNM delivered by "syringe only" technique [7]. Efficacy of the MAD for intranasal administration of drugs has been previously reported [7,8,15,16], as well as the MADgic® atomizer (Teleflex Incorporated, Wayne, Pennsylvania, United States) for airway topicalization [16]. 

We utilized a five-point sedation scale previously well described [13] to quantify sedation efficacy as classified by the blinded CRNA and RN evaluator. We also elected to have the parents participate in the evaluation of their own child’s level of distress in addition to directly-observed patient response and a blinded, trained RN evaluator.

Patient discomfort was directly measured by evaluating patient verbal complaints and patient signs of distress. Verbal complaints were in fact highest in the INM+L group at the time of administration compared to both INM and placebo (p=0.01). At one minute, verbal complaints were highest among the INM group but the INM+L group also reported more discomfort compared to the placebo group (p=0.03). In this study, it appears there was no benefit to adding lidocaine and in fact, it appears to worsen complaints at the time of administration. Our findings are consistent with O’Connell et al. who reported that pain and distress were comparable when intranasal lidocaine was administered before or concurrently with INM, but that intranasal lidocaine alone also caused significant pain and distress [17]. A combined dose would allow a single atomized dose compared to potential distress caused by two separate doses of Xylocaine (lidocaine) and midazolam. It would be interesting to compare a single dose versus multi-dose intranasal atomization in this patient population in future studies.

The addition of INM+L in this study was not associated with any epistaxis. Whether it contributed to subjective complaints of nasal stinging is unclear since this symptom was not specifically captured. INM+L was associated with a higher frequency of patient complaints at the time of administration but rapidly resolved by five minutes. Whether this was a surrogate for stinging is uncertain. No symptoms of neurotoxicity were documented in any patient during their hospital stay.

Sedation scores were favorable in the INM group compared to placebo at the time of induction of anesthesia. At 15 minutes post administration, both INM and INM+L groups had better sedation scores versus placebo. Interestingly, OBD was increased in the INM and INM+L groups compared to placebo as reported by RN observation and the INM+L may in fact exacerbate patient complaints compared to placebo. Sedation may be “adequate” in the INM group with no definitive advantage or disadvantage with INM+L. Nonetheless, both seem less favorable than placebo in affecting patient distress and comfort during intranasal sedation.

Based upon sedation scores in this study, at 10 minutes post administration, 100% of subjects were alert, calm, or drowsy; at 15 minutes, 7% of subjects were agitated. It would appear that the optimal time after administration for separation from the parents is 10 minutes. Unfortunately, we did not examine the response to sedation based upon sex or age group.

While no statistically significant differences were observed in delay of onset or discharge times, the INM+L group had a lower mean time to discharge compared to the INM group. If this difference was attributed to altering the bioavailability of INM because of adding lidocaine, a corresponding difference in levels of sedation and onset times might have been expected.

We acknowledge that this study was limited by the small number of subjects. As demonstrated in Figure 2, the patient population was difficult to reach, a factor further exacerbated by the attrition of an otolaryngologist during the course of study recruitment.

## Conclusions

In summary, the current study suggests that convenient “at the bedside” preparation of INM+L via MAD used as a premedication in children undergoing BMT resulted in more verbal complaints after administration than the placebo and also caused higher behavioral distress in the PACU. INM+L via atomization offers potential for rapid onset, precise delivery of the administered drug, and no effect on time to discharge. However, the addition of lidocaine as described in this study offers no clear advantage in patient comfort compared to INM alone and may statistically lower patient acceptance. Our data shows that at induction of anesthesia, INM premedication with the addition of lidocaine offers no definitive advantage over INM alone.

As a rural teaching hospital, our patient base was limited requiring a fairly broad age range. We also acknowledge this was a single-center study with a limited sample size. Further randomized and controlled studies with a larger study group and variable lidocaine dose would help elucidate the utility of adding lidocaine to INM. It is unclear if the addition of 4% lidocaine in a volume of 25% of the midazolam volume is efficacious in reducing side effects or enhancing patient acceptance.

## References

[1] Beeby DG, Hughes JO (1980). Behaviour of unsedated children in the anaesthetic room. Br J Anaesth.

[2] Bevan JC, Johnston C, Haig MJ (1990). Preoperative parental anxiety predicts behavioural and emotional responses to induction of anaesthesia in children. Can J Anaesth.

[3] Steward DJ (1989). Psychological preparation and premedication. Manual of Pediatric Anesthesia: the Hospital for Sick Children, Toronto, Canada.

[4] Laycock GJ, McNicol LR (1988). Hypoxaemia during induction of anaesthesia--an audit of children who underwent general anaesthesia for routine elective surgery. Anaesthesia.

[5] Raftery S, Warde D (1990). Oxygen saturation during inhalation induction with halothane and isoflurane in children: effect of premedication with rectal thiopentone. Br J Anaesth.

[6] Feld LH, Negus JB, White PF (1990). Oral midazolam preanesthetic medication in pediatric outpatients. Anesthesiology.

[7] Karl HW, Keifer AT, Rosenberger JL, Larach MG, Ruffle JM (1992). Comparison of the safety and efficacy of intranasal midazolam or sufentanil for preinduction of anesthesia in pediatric patients. Anesthesiology.

[8] Chiaretti A, Barone G, Rigante D (2011). Intranasal lidocaine and midazolam for procedural sedation in children. Arch Dis Child.

[9] Del Pizzo J, Callahan JM (2014). Intranasal medications in pediatric emergency medicine. Pediatr Emerg Care.

[10] Bhakta P, Ghosh BR, Roy M, Mukherjee G (2007). Evaluation of intranasal midazolam for preanasthetic paediatric patients. Indian J Anaesth.

[11] Akhlagh SH, Farbood A, Hadavi SM, Estabragh RR, Shahabifar R, Derakhshandeh S (2014). The effects of Midazolam pre-medication on gastric acidity and gastric contents in children prior to anesthesia. Euro J Exp Bio.

[12] Manley MC, Ransford NJ, Lewis DA, Thompson SA, Forbes M (2008). Retrospective audit of the efficacy and safety of the combined intranasal/intravenous midazolam sedation technique for the dental treatment of adults with learning disability. Br Dent J.

[13] Wilton NC, Leigh J, Rosen DR, Pandit UA (1988). Preanesthetic sedation of preschool children using intranasal midazolam. Anesthesiology.

[14] Haschke M, Suter K, Hofmann S (2010). Pharmacokinetics and pharmacodynamics of nasally delivered midazolam. Br J Clin Pharmacol.

[15] Lane RD, Schunk JE (2008). Atomized intranasal midazolam use for minor procedures in the pediatric emergency department. Pediatr Emerg Care.

[16] Xue FS, Yang QY, Liao X, Liu JH, Tong SY (2007). Topical anesthesia of the airway using fibreoptic bronchoscope and the MADgic atomizer in patients with predicted difficult intubation. Can J Anaesth.

[17] O'Connell NC, Woodward HA, Flores-Sanchez PL (2020). Comparison of preadministered and coadministered lidocaine for treating pain and distress associated with intranasal midazolam administration in children: a randomized clinical trial. J Am Coll Emerg Physicians Open.

